# Giving Everyone a Voice in Achieving a Just Transition in Welsh Domiciliary Care

**DOI:** 10.1093/geroni/igaf122.007

**Published:** 2025-12-31

**Authors:** Philip Taylor, Katharina Sarter

**Affiliations:** University of Warwick, Coventry, England, United Kingdom; Warwick Institute for Employment Research, Coventry, England, United Kingdom

## Abstract

Amid efforts to decarbonize society, the voices of socially disadvantaged community members, e.g. women low-paid workers, are often overlooked. Given the nature and growing significance of domiciliary care and the characteristics and size of its workforce, it represents a potentially valuable case study of involving communities in the co-design of solutions for a sector already undergoing significant transformation and now facing scrutiny as a major carbon emitter. This paper reports the preliminary findings of JUST-Systems, a UK-wide research initiative aimed at developing integrated systems approaches that position people at the center of efforts to accelerate action on decarbonization, local economies, and social justice. JUST-Systems seeks to identify bottlenecks and design better, more inclusive interventions by understanding the interactions between people, policies, and technologies. By adopting a systems approach, it may be possible to identify synergies and trade-offs between climate strategies and other priorities, such as jobs and social inclusion, focus on key obstacles and leverage points for change, and highlight what interventions can yield co-benefits. Welsh domiciliary care is one of the project’s test cases. This paper explores how systems thinking can aid in identifying synergies and trade-offs between climate strategies and the sector’s other policy priorities and challenges, emphasizing key barriers and leverage points for change. The paper reports on applying participatory action research methods and the Critical Systems Heuristics framework for reflective practice to reveal worker views around delivering domiciliary care through a decarbonization lens, identifying the opportunities and challenges in utilizing such techniques in facilitating worker voice.

